# Immunosuppressive treatment in autoimmune decompensated cirrhosis, when to say enough: A retrospective analysis

**DOI:** 10.1097/MD.0000000000041378

**Published:** 2025-02-07

**Authors:** Manuel Barbero, Stefania Burgos, Ignacio Roca, Lucia Navarro, Fernando Cairo

**Affiliations:** a Liver Transplant Unit, Hospital El Cruce de Florencio Varela, Buenos Aires, Argentina.

**Keywords:** autoimmune hepatitis, decompensated cirrhosis, immunosuppressive treatment, liver transplantation

## Abstract

Immunosuppressive therapy in decompensated cirrhotic patients with an indication for liver transplantation (LT) is controversial. This study aims to evaluate transplant-free survival rates in patients diagnosed with decompensated autoimmune hepatitis (AIH) with and without immunosuppressants (IS) treatment, and to identify predictors of mortality or LT. We retrospectively analyzed LT-evaluated consecutive patients with decompensated AIH at a single center, between 2013 and 2021. Patients were categorized into IS (IS Group) and non-IS (No IS Group). Survival curves for the 2 groups were evaluated using the Kaplan–Meier method, and differences were compared using the Log-rank test. Multiple regression analysis was conducted using Cox test. A total of 125 consecutive patients (mean age: 36.4 years; 81.6% female) were evaluated. The median transplant-free survival in the IS Group (72/125) were 22.6 months vs 6.57 months in the No IS Group (53/125) (*P* = .002). Cox-regression analysis revealed associations between moderate/severe ascites (hazard ratio [HR] = 2.37, 95% CI = 1.48–3.80, *P* = <.001) and MELD-Na (HR = 1.12, 95% CI = 1.08–1.16, *P* = <.001) with transplantation or death, while immunosuppression treatment acted protectively (HR = 0.55, 95% CI = 0.86–0.85, *P* = .008). Analyzing patients with MELD >22 (27/125) showed a loss of IS protective effect (OR = 0.45, 95% CI = 0.17–1.20, *P* = .11), and the presence of moderate/severe ascites increased the risk of death/LT (HR = 5.86, 95% CI = 1.26–27.2, *P* = .02). Autoimmune decompensated cirrhosis is associated with high overall mortality, and IS improves the transplant-free survival rate. In patients with MELD-Na >22, treatment ceases to be beneficial, especially if they have moderate/severe ascites. These patients, if receiving immunosuppression, may be disadvantaged in the possibility of accessing LT.

## 1. Introduction

Autoimmune hepatitis (AIH) is a disease with heterogeneous presentation, and in 1/3rd of patients, it presents with cirrhosis.^[1,2]^ Treatment with steroids and azathioprine has not only been shown to increase transplant-free survival rates in these patients but also to decrease fibrosis progression.^[3–7]^

Cirrhosis can be subdivided into compensated and decompensated types. It is well known that the survival rate differs depending on whether compensated or decompensated cirrhosis is present, as both stages must be considered as separate entities.^[8–10]^ Liver transplantation (LT) is an effective treatment for patients with AIH at the decompensated cirrhosis stage, with a posttransplant life expectancy similar to that of other etiologies.^[11,12]^

There are no doubts about the indications for immunosuppressive treatment in patients at the compensated cirrhosis stages.^[13,14]^ However, in decompensated cirrhosis with an indication for LT, the use of immunosuppressive treatment is controversial because of the risk of infections and the subsequent missed LT opportunity. The decision to initiate or continue immunosuppressive treatment in patients evaluated for LT is often challenging, because the predictors of treatment response at this stage of the disease are unknown.

Primary Objective:

- To evaluate transplant-free survival in patients diagnosed with decompensated cirrhosis of AIH etiology, with and without immunosuppressive treatment.

Secondary Objectives:

- To analyze the demographics and clinical presentation of patients with AIH evaluated for LT.
- Establish clinical, biochemical, and imaging factors predictive of mortality or LT in this segment of the population.
- To describe the prevalence and severity of infections in patients diagnosed with decompensated cirrhosis of AIH etiology with an indication for LT.
- To analyze a subgroup of severely ill patients to discern which group of patients no longer benefited from immunosuppressive treatment.

## 2. Methods

### 2.1. Patients

A retrospective analysis was conducted on patients evaluated for LT with a diagnosis of decompensated cirrhosis of AIH etiology at the El Cruce Hospital in Florencio Varela, Buenos Aires, from January 2013 to December 2021. In total, 125 consecutive patients were included in this study. The study was approved by the Ethics Committee of the El Cruce Hospital.

AIH was diagnosed according to the diagnostic criteria of the International Autoimmune Hepatitis Group (IAHG).^[15]^ Previous medical records were reviewed, and the history of steroid response was assessed. Histology was evaluated in patients for whom it was available. Patients with Patients with overlap syndrome and other causes of chronic liver disease were excluded from this study. Viral hepatitis (A, B, and C), MASLD, primary biliary cholangitis, Wilson disease, and α1-antitrypsin deficiency were ruled out by serology. A thorough investigation of alcohol consumption and drug toxicity was conducted. Abdominal ultrasound with Doppler of the splenoportal axis was performed in all patients, and contrast-enhanced triphasic computed tomography and/or Magnetic Resonance Imaging was performed to exclude hepatocellular carcinoma, vascular disorders, and biliary diseases.

The diagnosis of cirrhosis was confirmed by histological or unequivocal biochemical and imaging tests. Decompensation of cirrhosis was defined by the presence of clinical complications, such as esophageal varices, ascites, hepatic encephalopathy, hepatorenal syndrome, or impaired hepatic synthesis.^[8]^

### 2.2. Variables

Clinical, biochemical, immunological, and radiological data were also recorded. Age, sex, and previous episodes of decompensation were also evaluated. At the time of LT evaluation, alanine aminotransferase (ALT), aspartate aminotransferase (AST), alkaline phosphatase (ALP), gamma-glutamyl transferase, albumin, total bilirubin, international normalized ratio, prothrombin concentration, creatinine (Cr), urea, sodium (Na), platelet count, and hematocrit were assessed. Autoantibodies relevant to autoimmune liver disease were recorded, including antinuclear antibodies (ANA), anti-smooth muscle antibodies (ASMA), liver-kidney microsomal antibody type 1 (anti-LKM1), and antimitochondrial antibodies (AMA). ANA, ASMA, and AMA were analyzed using indirect immunofluorescence (IIF) in monkey liver sections, rat kidney, rat stomach tissue, and HEp-2 cells. LKM1 antibody levels were examined by immunoblotting. Serum levels of immunoglobulins, including IgG, were recorded. The presence of mild, moderate, or severe ascites was documented using ultrasonography, computed tomography, and MRI. The MELD-Na score was calculated for all patients at the time of the transplant assessment.

### 2.3. Treatment

Of the 125 patients included in this study, 72 were treated with corticosteroids, azathioprine, and/or mycophenolate. The decision to administer treatment, as well as the dose and duration of treatment from the pretransplant evaluation, were determined by the treating physicians based on the patients’ evolution. Patients were divided into an immunosuppression group (IS Group) and a no immunosuppression group (No IS Group). The first group included all patients who received any dose of steroids and/or azathioprine for up to 30 days before LT evaluation and/or were on treatment at the time of evaluation.

### 2.4. Follow-up and outcome

Treatment records were reassessed at 30, 90, and 365 days post-LT evaluation. The presence of infections was scrutinized from the pre-LT evaluation and persisted throughout the follow-up period at our center. The dates of LT and death were recorded. For patients who were still alive, the data were recorded as of August 2023. Patients who had not undergone medical checkups for over 6 months were contacted by phone to reassess their clinical situation.

### 2.5. Statistical analysis

Continuous variables with a normal distribution are presented as mean and standard deviation. Non-parametric distributions are described as medians and interquartile ranges (IQRs). Categorical variables are expressed as frequencies and percentages. The t-test and Mann–Whitney *U* test were used to compare continuous variables. Categorical variable analysis was performed using the chi-squared test or Fisher exact test.

Survival curves for the 2 groups were evaluated using the Kaplan–Meier method, and differences were compared using the Log-rank test. Multiple regression analysis was conducted using Cox test. All analyses were performed using R statistical software version 4.2.1.13. Statistical significance was set at *P* < .05.

## 3. Results

### 3.1. Diagnosis and general characteristics of autoimmune hepatitis in decompensated cirrhosis stage

During the study period, 1321 patients with decompensated cirrhosis were examined for LT, of which 125 (9.5%) had AIH etiology and were included in the study. The mean age was 36.4 years, and 81.6% of the patients were female.

Of the 125 patients, 97 (77.6%) tested positive for 1 or more autoantibodies. ANA and ASMA were equal to or >1/80 in 68% and 46.6% of patients with AIH, respectively; anti-LKM1 was present in only 4 patients. Histological analysis was performed in 43 patients with diagnostic uncertainties based on previous biopsies or explant analyses in transplanted patients. Among the 28 patients with negative autoantibodies, the diagnosis of AIH was based on elevated levels of γ-globulin, histological analysis when available (14/28), response to steroids, and ruling out other causes of chronic liver disease. The median γ-globulin was 2.05 g/dL (IQR 1.6–2.9 g/dL).

### 3.2. Characteristics of treated and untreated patients with immunosuppression

Of the 125 patients, 72 (57.6%) received immunosuppression at the time of transplantation assessment (IS Group) and 53 (42.4%) did not receive any immunosuppression (No IS Group). The mean MELD-Na scores in the IS Group was 16.5 and 19.4 in the No IS Group, a statistically significant difference (*P* = .002). The presence of moderate-to-severe ascites was 23.6% in the immunosuppression-treated group and 50.9% in the untreated group (*P* = .002). The remaining evaluated variables are presented in Table 1.

**Table 1:** Clinical patient characteristics.

Variables	No IS group (N = 53)	IS group (N = 72)	*P*-value
Pre-LT evaluation variables			
Age (yr) (median, IQR)	38 (28–52)	29.00 (21.75–42.25)	.016 (3)
Male gender (%)	9 (17.0%)	14 (19.4%)	.725 (2)
MELD-Na (median, IQR)	19 (15–24)	15 (11.00–20.25)	.015 (1)
AST (U/L)	70 (49–119)	93 (46.00–186.00)	.359 (3)
ALT (U/L)	49 (28.00, 86.00)	84.5 (45.00, 202.75)	<.001 (3)
BT (mg/dL)	3.23 (1.92, 7.41)	2.37 (1.47, 4.82)	.231 (3)
INR	1.57 (1.22, 1.88)	1.35 (1.24, 1.64)	.094 (3)
Sodium (mmol/L)	136 (133–139)	139 (136.00, 140.00)	.008 (4)
ALP (UI/L)	182 (131.00–250.00)	177.5 (115.75, 251.00)	.795 (3)
Platelets (K/µL)	69.000 (55.000–92.000)	70.500 (48.750–107.250)	.824 (3)
Creatinine (mg/dL)	0.65 (0.57–0.79)	0.60 (0.53–0.74)	.816 (4)
Albumin (g/dL)	2.57 (2.17–3.26)	2.79 (2.31–3.31)	.189 (4)
Moderate/severe ascites, n (%)	42 (79.2%)	38 (52.8%)	.002 (2)
Hepatic encephalopathy, n (%)	21 (39.6%)	31 (43.1%)	.700 (2)
Esophageal varices, n (%)	39 (73.6%)	56 (77.8%)	.588 (2)
Spontaneous bacterial peritonitis, n (%)	10 (18.9%)	12 (16.7%)	.749 (2)
Non-PBE infections, n (%)	11 (20.8%)	18 (25.0%)	.578 (2)
Steroid dose	0 (0–0)	8 (4.0–20.0)	<.001 (4)
Autoantibodies (%)	41 (77.4%)	56 (78.9%)	.840 (2)
ANA, n (≥1.40) (%)	35 (66.0%)	50 (69.4%)	.687 (2)
ASMA, n (≥1.40) (%)	22 (41.5%)	36 (50.0%)	.347 (2)
Anti-LKM1, n (%)	3 (5.7%)	0 (0.0%)	.088 (2)
IgG level	2363.50 (1856–2751)	2123.50 (1596–2741)	.658 (4)
Ceruloplasmin	25 (21–30)	25 (21–28)	.779 (4)
Liver biopsy	18 (34.0%)	25 (34.7%)	.930 (2)
Post–pre-LT evaluation variables			
Death	12 (22.6%)	20 (27.8%)	.516 (2)
Liver transplantation	32 (60.4%)	27 (37.5%)	.011 (2)
Infections, n (%)	18 (34.6%)	25 (34.7%)	.990 (2)

(1): median test; (2): Pearson chi-squared test; (3): Kruskal–Wallis rank sum test; (4): Linear Model ANOVA.

ALP = alkaline phosphatase, ALT = alanine aminotransferase, ANA = antinuclear antibody, Anti-LKM1 = liver-kidney microsomal type 1 antibodies, ASMA = anti-smooth muscle antibody, AST = aspartate aminotransferase, BT = total bilirubin, EPS = portosystemic encephalopathy, IgG = immunoglobulin G, IgG = immunoglobulin G, INR = international normalized ratio, IQR = interquartile range, SBP = spontaneous bacterial peritonitis.

### 3.3. Treatment

Of the 72 patients in the IS group, 35 received corticosteroids only, 32 received corticosteroids and azathioprine, 4 received corticosteroids and mycophenolate, and only 1 patient received azathioprine alone. The median dose of prednisone treatment in the IS group was 8 mg (IQR 4–20), the dose of azathioprine was 50 mg for all patients, and the dose of mycophenolate was 1000 mg/day.

### 3.4. Analysis of survival and associated prognostic factors

**Figure 1.**
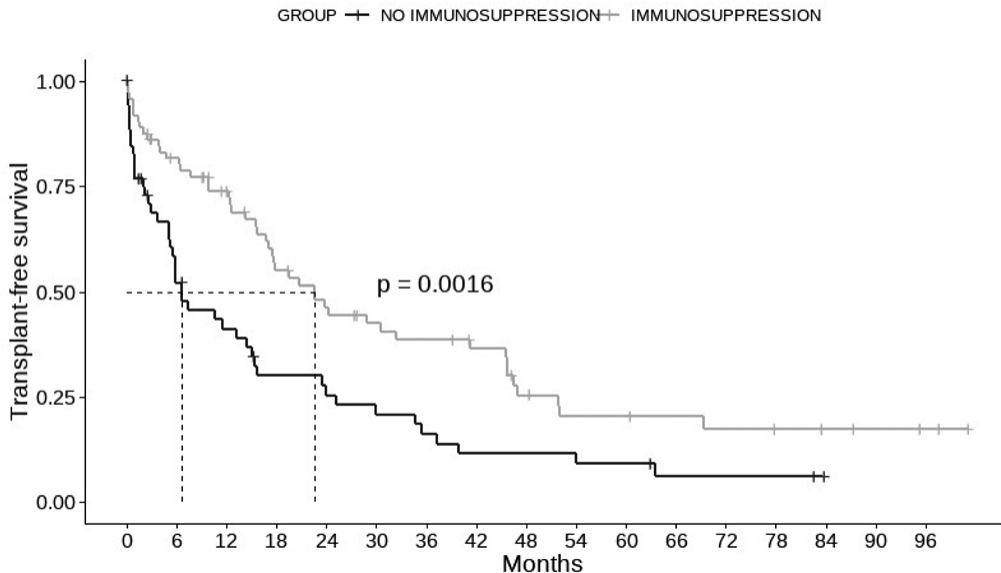
Kaplan–Meier method and Mantel–Cox test. Analysis of transplant-free survival comparing the treated group vs the untreated group.

The median transplant-free survival was higher in the immunosuppressed group than in the untreated group (22.6 vs 6.57 months), and this difference was statistically significant (*P* = .0016) (Fig. 1). Univariate analysis was conducted to evaluate the variables associated with transplant-free survival (Table 2). A multiple regression analysis was then performed, and the variables associated with the need for transplantation or death were moderate-to-severe ascites (HR = 2.37, 95% CI = 1.48–3.80, *P* < .001) and the MELD-Na score (HR = 1.12, 95% CI = 1.08–1.16, *P* < .001), while IS treatment acted as a protective factor (HR = 0.55, 95% CI = 0.86–0.85, *P* = .008) (Table 3).

**Table 2:** Univariate analysis.

Variables	Transplant-free survival (n = 34)	Death/LT (n = 91)	Valor *P*
Age (yr) (median, IQR)	32.5 (24.50–51.75)	32 (25.00–44.50)	.957 (1)
Male gender (%)	6 (17.6%)	17 (18.7%)	.894 (2)
MELD-Na (median, IQR)	13 (10–17)	18 (13–23.50)	.005 (1)
AST (U/L)	100 (44.50–233)	72 (48.50–127.50)	.466 (3)
ALT (U/L)	91.5 (42.50–305.25)	60 (35–109.50)	.045 (3)
BT (mg/dL)	1.97 (1.12–2.63)	3.50 (1.78–7.39)	.002 (3)
INR	1.31 (1.16–1.53)	1.49 (1.24–1.81)	.018 (3)
Sodium (mmol/L)	139 (137.25–140.75)	137 (134–140)	.019 (4)
ALP (UI/L)	181.5 (129.25–254.75)	177 (127–249.50)	.881 (3)
Platelets (K/µL)	84.500 (62.000–154.500)	65.000 (41.000–81.500)	<.001 (3)
Creatinine (mg/dL)	0.62 (0.56–0.72)	0.62 (0.54–0.81)	.250 (4)
Albumin (g/dL)	3.12 (2.47–3.42)	2.61 (2.25–3.23)	.020 (4)
Moderate/severe ascites, n (%)	17 (50.0%)	63 (69.2%)	.046 (2)
Hepatic encephalopathy, n (%)	10 (29.4%)	42 (46.2%)	.091 (2)
Esophageal varices, n (%)	22 (64.7%)	73 (80.2%)	.071 (2)
SBP, n (%)	2 (5.9%)	20 (22.0%)	.035 (2)
Non-SBP infections, n (%)	4 (11.8%)	25 (27.5%)	.064 (2)
IS treatment, n (%)	25 (73.5%)	47 (51.6%)	.028 (2)
Autoantibodies (%)	28 (82.4%)	69 (76.7%)	.494 (2)
ANA, n (≥1.40) (%)	26 (76.5%)	59 (64.8%)	.215 (2)
ASMA, n (≥1.40) n (%)	18 (52.9%)	40 (44.0%)	.370 (2)
Anti-LKM1, n (%)	1 (2.9%)	2 (2.2%)	.806 (2)
IgG level	2730 (1708–3400)	2200 (1646–2598)	.004 (4)
Ceruloplasmin	26 (21.25–30)	25 (21–28)	.447 (4)
Infections after pre-LT evaluation	39 (43.3%)	51 (56.7%)	<.001 (2)

(1): median test; (2): Pearson chi-squared test; (3): Kruskal–Wallis rank sum test; (4): linear model ANOVA.

ALP = alkaline phosphatase, ALT = alanine aminotransferase, ANA = antinuclear antibody, Anti-LKM1 = liver-kidney microsomal type 1 antibodies, ASMA = anti-smooth muscle antibody, AST = aspartate aminotransferase, BT = total bilirubin, EPS = portosystemic encephalopathy, IgG = immunoglobulin G, IgG = immunoglobulin G, INR = international normalized ratio, IQR = interquartile range, SBP = spontaneous bacterial peritonitis.

**Table 3:** Multivariate analysis.

Characteristic	HR	95% CI	*P*-value
Treatment	0.55	0.36 to 0.85	.008
MELD-Na	1.12	1.08 to 1.16	<.001
Moderate/severe ascites	2.37	1.48 to 3.80	<.001

### 3.5. Infections

The number of patients who experienced at least 1 infection during their evolution was 25 (34.7%) in the IS group and 18 (34.6%) in the No IS group, and this difference was not statistically significant (*P* = .9). Among the 25 IS patients who had at least 1 infection, 32 infection episodes were observed: 7 community-acquired pneumonia (CAP), 7 urinary tract infections (UTI), 6 spontaneous bacteremias, 6 skin and soft-tissue infections (SSTI), 5 spontaneous bacterial peritonitis (SBP), and 1 meningitis. Of the 32 episodes, 9 were due to multi-drug-resistant organisms and 2 were fungal. Eight patients died of infection. Among the 18 patients in the No IS group, 23 infections were recorded: 7 CAP, 6 SBP, 5 UTI, 2 SSTI, and 3 spontaneous bacteremias. Only 1 multi-drug-resistant infection and no fungal infections were reported, and 3 patients died due to infection.

### 3.6. Patients with MELD-Na > 22

Of the 125 evaluated patients, 27 (21.6%) had an MELD-Na value > 22, and a sub-analysis of this more severe population was conducted. Of the 27 patients, 12 (44.4%) received IS, and 15 (55.6%) did not. When performing a multivariate analysis to predict variables associated with the need for transplantation or death, it was observed that having moderate-to-severe ascites in this subpopulation continued to be a poor prognostic factor (HR = 5.86, 95% CI = 1.26–27.2, *P* = 0.02). However, IS treatment lost its protective effect (OR = 0.45, 95% CI = 0.17–1.20, *P* = 0.11) (Table 4).

**Table 4:** Multivariate analysis in patient population with MELD-Na > 22.

Characteristic	HR	95% CI	*P*-value
Treatment	0.45	0.17 to 1.20	.11
Moderate/severe ascites	5.86	1.26 to 27.2	.024

It is noteworthy that in the subpopulation of patients with MELD-Na > 22, 9 patients (81.8%) from the No IS group underwent transplantation, and 2 (18%) died; in the IS group, only 3 (37.5%) underwent transplantation, and 4 (50%) died without it.

## 4. Discussion

This study presents the largest series of patients with decompensated cirrhosis reported to date. Autoimmune hepatitis exhibits a varied global distribution in terms of both incidence and prevalence. A recent systematic review and meta-analysis estimated the global pooled incidence at 1.28 cases per 100,000 inhabitant-years and a prevalence of 17.44 cases per 100,000 persons. While regional variations exist, Europe and North America generally report higher prevalence than Asia.^[16,17]^ In Latin America, while comprehensive data remains limited, studies suggest that AIH is not rare, according to reports, autoimmune hepatitis accounts for 4% to 6% of adult liver transplants in Peru and is the second leading cause of acute liver failure in Argentina.^[18,19]^ Notably, in the present study, AIH was identified as the underlying etiology in 16.5% of cases of decompensated cirrhosis requiring LT.

AIH may present at any age and in all ethnic groups. In most studies, a bimodal age pattern at presentation has been reported with 1 peak during childhood/teenage years and another in middle age between the 4th and 6th decade of life.^[13]^ The median age of diagnosis for autoimmune hepatitis has shown a clear upward trend over recent decades. For instance, an Italian study observed median ages at diagnosis increasing progressively from 24 years in the 1980s to 52 years in the 2010s.^[20]^ This aging trend is relevant to the presentation of AIH, as cirrhosis at presentation is notably more frequent in older patients. One study found that individuals aged 60 or older were more than 3 times as likely to present with cirrhosis compared to those aged 30 or younger (33% vs 10%).^[21]^ In our study, the mean age was 36.4 years, which is lower than that typically reported in the literature for patients presenting with cirrhosis. One possible explanation could be that all the patients in our study were referred for LT.

In our cohort, more than 20% of patients did not test positive for autoantibodies. The primary criterion employed in our study was the simplified diagnostic criteria established by the IAHG,^[15]^ as many participants had already received a prior diagnosis of autoimmune hepatitis and were, in most cases, receiving immunosuppressive therapy. Additional factors, such as patients’ medical histories, previous liver biopsies, explanted livers from transplant recipients, and response to steroid treatment, were also considered in confirming the diagnosis of autoimmune hepatitis. It remains undetermined whether the proportion of positive autoantibodies and serum gamma globulin levels are lower in patients with decompensated cirrhosis, as seen in those with acute AIH presentations.^[22,23]^

The prognosis for AIH presenting with cirrhosis is generally poor, with a high risk of progressing to death or transplantation in the short to medium term.^[24,25]^ The presence of cirrhosis at the time of autoimmune hepatitis diagnosis significantly impacts long-term outcomes. A 10-year survival rate of 61.9% has been reported for patients presenting with cirrhosis at diagnosis, sharply contrasting with the 94.0% survival observed in those without cirrhosis.^[26]^

However, existing literature on the prognosis of patients with autoimmune decompensated cirrhosis is limited. In the present cohort, the median transplant-free survival time was only 15.7 months.

Immunosuppressive therapy in AIH has been shown to improve patient survival, as demonstrated by Cook et al from studies published over 50 years ago,^[3]^ as well as in its efficacy in reducing fibrosis stages 4 to 7. However, controversies persist, especially in patients with decompensated cirrhosis, primarily because of the potentially increased risk of infections in this subset of patients.^[27–29]^ In our series, immunosuppressive treatment enhanced transplant-free survival across the evaluated population, with a median survival of 22.6 vs 6.57 months, this difference was statistically significant. Nevertheless, it is essential to note that the severity in the untreated group at the time of evaluation was more pronounced, as evidenced by higher MELD-Na scores (19.4 vs 16.5) and a greater proportion of patients with severe ascites (50.9% vs 23.6%), both of which were statistically significant differences.

Sharma et al published a study involving 94 patients with cirrhosis of autoimmune etiology (62 diagnosed with decompensated cirrhosis) and identified the MELD score and the presence of ascites as predictors of poor prognosis in survival analysis. Transplant-free survival varied significantly based on ascites severity, with rates of 25.4% for gross ascites, 74.6% for mild/no ascites, and 96.9% for compensated cirrhosis.^[30]^ In our study of 125 patients with decompensated cirrhosis, multivariate analysis revealed that the 2 factors associated with death or transplantation were the presence of severe ascites (hazard ratio [HR] = 2.37) and the MELD-Na value (HR = 1.12). A noteworthy observation is that immunosuppressive treatment acted as a protective factor (HR = 0.55) across the entire study population.

The negative impact of bacterial infections on cirrhosis is clinically relevant at any stage of liver disease but is particularly pronounced in decompensated cirrhosis of any etiology. This concern is exacerbated by the increase in infections caused by multi-drug-resistant organisms.^[27]^ The use of corticosteroids in other liver diseases, such as severe alcoholic hepatitis, has been shown to increase the risk of infections. Pulmonary infections were reported in a substantial proportion (40.3%) of patients receiving corticosteroids for severe alcoholic hepatitis. Bacterial infections, particularly with Enterococcus species, are also common in this setting. Furthermore, corticosteroids are associated with an increased frequency of fungal infections, especially with Candida species. This heightened risk of infection underscores the importance of careful consideration when using corticosteroids in patients with liver disease, particularly those with decompensated cirrhosis.^[31–33]^ In our study, although there was no statistically significant difference in the number of patients presenting with infections between both groups, the immunosuppressed (IS) group experienced more frequent episodes of infections, multi-drug resistant and fungal infections, and a higher number of deaths due to infections.

Liver transplantation remains the definitive treatment for patients with decompensated cirrhosis of autoimmune etiology, with long-term survival rates similar to those of patients with other etiologies.^[11]^ Conversely, Wang et al reported a subset of 40 of 64 patients who achieved cirrhosis recompensation with steroid treatment, while 15 remained decompensated, and 9 experienced liver-related death or transplantation.^[34]^ To identify patients who might not benefit from immunosuppressive therapy, we analyzed a subgroup of patients with MELD-Na > 22. At this cutoff point, treatment lost its protective effect in multivariate analysis, while the presence of moderate-to-severe ascites remained a prognostic factor in this subset of severely ill patients. In this regard. These patients with immunosuppressive treatment may even lose the chance of transplantation due to the decrease of the MELD score and evolve into “purgatory,” as seen in patients with hepatitis C treatment.

All analyses in our study focused on assessing transplant-free survival, considering transplantation to be a competing event for patient death. However, this approach might overlook a crucial aspect of the decision-making process in critically ill patients. Some patients may lose the chance for transplantation due to severe infections resulting from immunosuppressive treatment. Analysis of the subgroup of patients with MELD-Na > 22 revealed that 81.8% of the No IS group underwent transplantation, with only 18% dying, whereas in the IS group, only 37.5% underwent transplantation, and 50% died. The limited number of patients precluded statistical analysis; however, this remains an important consideration for future research.

Our study had several limitations. First, it was a retrospective design, and a significant number of patients were on immunosuppression upon admission, complicating the definitive diagnosis of autoimmune hepatitis using standardized criteria. The decision regarding which patients received immunosuppression prior to the pretransplant evaluation was not consistently made by the medical team conducting the study, leading to inconsistent criteria. The absence of randomization prevented additional analyses. However, randomization of patients to receive immunosuppression in this scenario is ethically controversial. Conversely, we believe that the strength of our study lies in the number of patients included from a rare disease population at an understudied stage. We emphasize the importance of conducting a prospective, multicenter study to validate our findings.

## 5. Conclusion

Decompensated cirrhosis of autoimmune etiology is associated with high overall mortality. Immunosuppressive treatment improves transplant-free survival. However, due to the increased risk of infection in patients with MELD-Na > 22, our study suggests that treatment loses its benefit, especially when the patient exhibits moderate-to-severe ascites. Critically ill patients receiving immunosuppression may have compromised their potential access to LT. Careful patient selection and close monitoring for infections are crucial when considering immunosuppressive therapy in this population. Further prospective studies are warranted to identify which patients with decompensated autoimmune hepatitis-related cirrhosis are most likely to benefit from immunosuppressive therapy and to develop tailored treatment strategies that minimize the risk of complications.

## Author contributions

**Data curation:** Stefania Burgos, Lucia Navarro.

**Formal analysis:** Ignacio Roca.

**Methodology:** Ignacio Roca, Manuel Barbero.

**Supervision:** Fernando Cairo.

**Writing – original draft:** Manuel Barbero.

**Visualization:** Lucia Navarro.
